# Supplementary material for: Preparation of Magnetic–Luminescent Bifunctional Rapeseed Pod-Like Drug Delivery System for Sequential Release of Dual Drugs
Source: Pharmaceutics. 2021 Jul 22;13(8):1116. doi: 10.3390/pharmaceutics13081116 (PMC8398606; doi:10.3390/pharmaceutics13081116)
Supplement: Supplementary file 1 [file pharmaceutics-13-01116-s001.zip › pharmaceutics-1258728-supplementary.pdf]

pharmaceutics

Supplementary Materials: Preparation of Magnetic-Luminescent Bifunctional Rapeseed Pod-like Drug Delivery System for Sequential Release of Dual Drugs

Junwei Xu , Yunxue Jia , Meili Liu , Xuenan Gu , Ping Li , and Yubo Fan

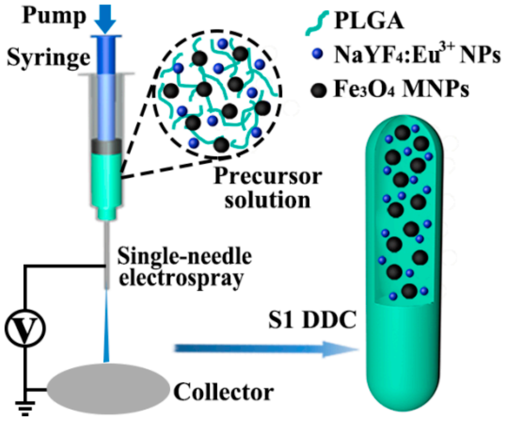

Figure S1. Schematic illustration of preparing magnetic-fluorescent bifunctional rapeseed pod-like [$NaYF_4$:Eu^{3+}/Fe_3O_4/PLGA] DDCs (denoted as S1) via single-needle electrospray.

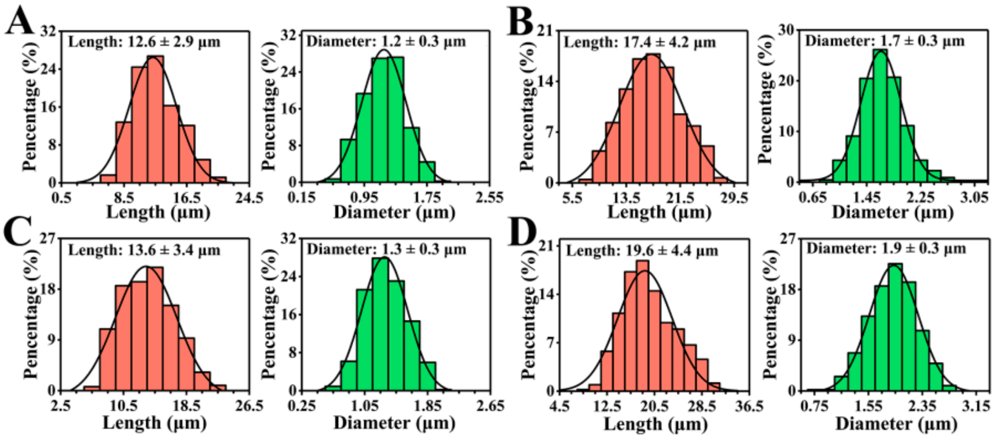

Figure S2. Length and diameter distributions of DDCs: (**A**) S1 DDCs, (**B**) D1 DDCs, (**C**) S2 DDCs, (**D**) D2 DDCs; for each kind of DDCs, the length and diameter of at least 400 microparticles' were measured by using the software of image J 1.48v.

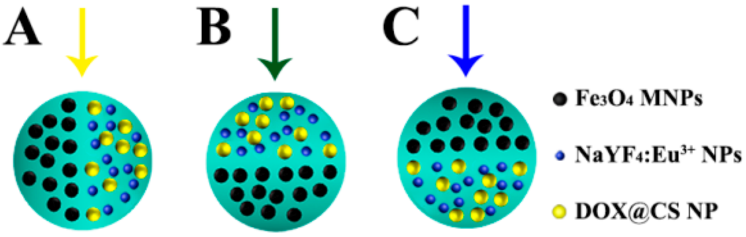

Figure S3. Three typical cross-sectional schematic diagrams of D2 DDC collected on the collector: (**A**) double chambers were left/right distributed; (**B**) and (**C**) double chambers were up/down distributed, (**B**) the chamber embedded with $NaYF_4$:Eu^{3+} NPs and CS NPs facing upward and (**C**) the chamber containing Fe_3O_4 MNPs facing upward.

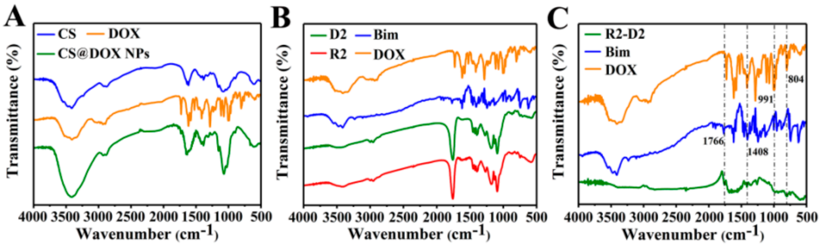

Figure S4. (**A**) FTIR spectra of CS, DOX and DOX@CS NPs; the characteristic peaks at 1733 and 1417 cm^{-1} belong to DOX and the characteristic peaks in the FTIR spectra of DOX@CS NPs at 3421 and 1064 cm^{-1} were significantly enhanced compared to CS. (**B**) FTIR spectra of Bim, DOX, D2 DDCs and R2 DDSs; (**C**) FTIR spectra of Bim and DOX, and infrared subtractive spectrum analysis of R2 DDSs and D2 DDCs; the characteristic peaks at 1766 cm^{-1} corresponded to the stretching vibration of C=N in Bim, at 1408 cm^{-1} indicated the vibration of C-H on the benzene ring of DOX or Bim, and at 991 and 804 cm^{-1} attributed to the vibration of C-C=O and benzene ring in DOX, respectively.
